# Supplementary material for: Are carbon emissions trading and green financial instruments synergistic? -Comprehensive quantitative research based on content analysis
Source: PLoS One. 2024 Mar 7;19(3):e0298601. doi: 10.1371/journal.pone.0298601 (PMC10919601; doi:10.1371/journal.pone.0298601)
Supplement: S1 Data — (ZIP) [file pone.0298601.s001.zip › Data/地级市Z_F工作报告.zip!/╕ú╜¿/╚2├≈╩╨/└┤╫╘╣1⁄2╓┌║┼í╛┬φ┐╦╩2╛▌═°í┐_2004.docx]

各位代表：

　　现在，我代表市人民政府向大会报告政府工作，请予审议，并请市政协各位委员和其他列席人员提出意见。

　　一、届首之年实现良好开局

　　2003年是本届政府届首之年。面对突如其来的非典疫情和历史罕见的持续干旱，面对激烈的竞争和繁重的改革发展稳定任务，我市各级政府在省委、省政府和市委的坚强领导下，以“三个代表”重要思想为指导，全面贯彻党的十六大、十六届三中全会精神，认真执行市十届人大一次会议决议，团结和依靠全市人民，坚持以发展为第一要务，盯住发展，谋划发展，致力发展；坚持解放思想，实事求是，与时俱进，大力创新体制，增强发展活力；坚持经济社会统筹兼顾，改革发展稳定互相促进，三个文明建设协调推进；坚持以人为本，转变职能，改进作风，努力为人民服务，建设对人民负责的政府。经过全市上下共同努力，较好地完成了市十届人大一次会议确定的各项目标任务，国民经济和社会各项事业取得新成绩。

　　经济保持较快增长。围绕实施项目带动战略、加快发展县域经济、壮大非公有制经济，与时俱进推出新举措，制定配套政策措施，突出解决影响三明发展的瓶颈制约和体制障碍，凝聚加快发展合力，推进经济扩总量、增后劲。初步统计，全年完成国内生产总值321.64亿元，增长10%。农林牧渔业总产值120.8亿元，增长4.4%；规模以上工业总产值237.44亿元，增长17.8%。全社会固定资产投资100.52亿元，增长29.4%。社会消费品零售总额107.05亿元，增长8.9%。居民消费价格总指数为101.6%。地方级财政一般预算收入15.13亿元，同口径增长18.8%。

　　抗非典抗大旱经受考验。去年上半年，在我市出现全省首例输入性非典患者的严峻形势下，我们靠前指挥、沉着应对、科学防治，按照一手抓防治非典不动摇、一手抓经济建设不放松的要求，建立监控机制，构筑防护网络，实现了无二代病例、无死亡病例、无医务人员感染的“三无”目标，保障了全市人民的健康安全，体现了抗击非典的实力、能力与合力。在防治非典的同时，我市又遭遇严重的特大旱灾，持续时间长、受灾面广，全市上下迎难而上，齐心协力，科学调度，合力抗灾，努力把灾害损失减少到最低程度，基本保证了全市城乡居民的正常生活，基本保证了全市经济的持续发展。

　　经济结构调整步伐加快。努力在加快发展中优化结构，在优化结构中提高经济运行的质量和效益。第二产业比重上升2.5个百分点，机械装备、林产加工、纺织、冶金等产业快速增长，生物制药、绿色产业等新兴产业开始起步。三明高新技术产业开发区金沙园、尼葛园和县级工业小区加快开发建设。规模以上工业对经济增长的贡献率达到37.3%，经济效益综合指数比上年提高20.7个百分点，实现利润增长1.08倍。农业龙头企业发展加快，50家省、市级农业产业化龙头企业建立基地520.93万亩，联系带动农户35.2万户，农村劳动力继续向二、三产业有序转移。商贸、旅游等服务业稳步发展。县域经济发展格局初步形成，特色经济亮点增多，活力逐步显现。

　　项目带动取得明显成效。开展“抓项目、增后劲、促发展”竞赛活动，创新项目工作载体和机制，调动了各级各部门抓项目的积极性，促进了固定资产投资的较快增长。45项市级在建重点项目已有19项竣工投产或部分投产。京福高速公路三明段完成投资23.1亿元，增长43.6%。泉三高速公路项目建议书获国务院批准，工程可行性研究报告已上报国家发改委和交通部。三明至宁化、永安至武平高速公路完成路线走向规划方案编制。交战公路建设第一期310公里进入扫尾阶段。农村公路开工建设935公里，建成水泥路面490公里，完成投资1.98亿元，增长3.7倍。街面水电站和一批中小型电站加快建设，城乡电网改造取得进展。

　　改革开放扎实推进。加快体制改革和机制创新，参与构筑三条战略通道，增强经济发展活力。一批国有企业通过深化改革焕发生机，雪津（三明）啤酒有限公司等企业优化重组后加快发展，立丰印染有限公司等企业实现国有资本退出和股权结构调整，三明标准件厂等企业破产重组进展顺利。简政放权力度加大，79项经济社会管理审批权限下放到10个县（市），49项审批权限下放到梅列、三元两区。市、区两级财政体制调整逐步到位，农村税费改革试点工作深入实施，集体林权制度改革进一步深化。非公有制经济快速发展，私营企业达到4789户，注册资金58.48亿元，分别增长33.5%和101.3%；个私经济上缴税收8.4亿元，增长25.2%。联内引外取得实效，实际利用外商直接投资按同比口径为7286万美元，增长19.3%；完成出口总值1.45亿美元，增长22.5%；已有6家企业到境外投资，出境劳务9768人；内联协作引进资金12.16亿元，增长30%。

　　人民生活得到改善。重视解决“三农”问题，做好就业再就业工作，加快社会保障体系建设，加大扶贫帮困力度，努力提高人民生活水平。城镇居民人均可支配收入9267元，增长7.2%；农民人均纯收入3556元，增长4.4%。全市金融机构人民币各项存款余额243.73亿元，增长20.8%；人民币各项贷款余额189.4亿元，增长24.6%。新增城镇就业30751人，下岗失业人员再就业13950人，城镇登记失业率为5.91%。养老保险、失业保险、医疗保险的覆盖率分别达到94.3%、98%和82%，按时足额发放企业离退休人员基本养老金和失业人员失业金。城市低保基本做到“应保尽保”，农村低保逐步展开。革命老区、贫困山区和少数民族地区群众的生产生活条件逐步改善，“造福工程”完成搬迁任务1419 人。年初确定的为民办实事项目基本完成。城市近期建设规划得到完善，城乡建设步伐加快，房地产投资增长21.3%，一批乡镇小区被列为省级村镇试点小区。完成了市区文化巷、绿岩等占道市场综合整治，建设、改造了一批小区路网等配套设施。

　　社会事业稳步发展。贯彻《公民道德建设实施纲要》，深入开展群众性精神文明创建活动，创文明社区、满意窗口、小康村镇和军警民共建活动取得成效，城乡文明程度逐步提高。科技创新体系建设全面推进，13项科技成果获2003年度省科学技术奖，开展产学研合作项目918项，实施国家技术创新工程区域试点工作和沙溪沿岸星火技术产业带建设成效明显。优化人才发展环境，深化人事制度改革，加大人才培养和引进力度，人才队伍建设进一步加强。“两基”成果巩固提高，素质教育和课程改革扎实推进，“名校”、“名师”工程取得成效；高等教育、职业教育、成人教育发展步伐加快，三明学院筹建工作进展顺利，创建了一批国家、省级重点中等职业学校。公共卫生体系建设进一步完善，预警和应急机制逐步建立，农村卫生工作加强，国家卫生城的成果得到巩固。群众文化活动广泛开展，精品创作取得成果，文化市场进一步规范，新闻出版工作管理有序。广播电视节目创作和传输水平有新的提高，一批节目在国家、省级比赛中获得较好名次。全民健身活动深入开展，竞技体育取得成绩，老年体育继续发展。人口与计划生育工作实现省下达的责任目标，人口自然增长率为4.8‰。国土资源保护开发力度加大，土地和矿业市场逐步健全，耕地占补平衡，初步实现矿业开发的规模化、集约化经营。生态建设和环境保护稳步推进，主要污染物排放总量得到控制。外事、侨务、民政、老区、地方志、民族、宗教、社会科学等工作取得新的成绩，老龄、妇女、儿童、残疾人和关心下一代等工作不断加强。国防动员、民兵预备役、人民防空等工作扎实开展，“双拥”共建活动深化，第四次获得“全国双拥模范城”称号。

　　发展环境逐步优化。认真执行人大及其常委会的决议、决定，自觉接受法律监督和工作监督，办好人大代表建议和意见。积极同政协进行民主协商，主动接受民主监督，办好政协提案。坚持依法行政和廉洁从政，深入开展反腐倡廉，不断提高行政能力和水平。继续深入开展“三减（简）一提高”活动，深化行政审批制度改革，设立市行政服务中心，项目审批提速。推行政务公开，强化效能监督和效能告诫，优化行政服务环境。推进基层民主建设，群众自治组织作用得到发挥。大力整顿和规范市场经济秩序，狠抓安全生产事故防范，强化社会治安综合治理，加强国家安全工作，做好群众来信来访和各类矛盾纠纷排查调处工作，保持了社会安定稳定，在全省9个设区市落实省市综治领导责任书综合考评中获总分第二名。

　　回顾过去一年，成绩来之不易。这些成绩的取得离不开省委、省政府的亲切关怀和正确领导，是在历届市委、市政府打下的坚实基础上，全市人民辛勤劳动、团结奋斗的结果；是与市人大及其常委会依法监督和支持，市政协政治协商、民主监督、参政议政，各民主党派、工商联、无党派人士、人民团体积极参与，中央、省属驻明单位、驻明部队和武警官兵、离退休老干部的大力支持，香港特别行政区同胞、澳门特别行政区同胞、台湾同胞、海外侨胞、国外友好人士和来明投资者的真诚合作分不开的。在此，我代表市人民政府，向所有关心和为三明改革发展稳定作出贡献的同志们、同胞们、朋友们表示衷心的感谢和崇高的敬意！

　　在总结成绩的同时，我们也清醒地看到存在的矛盾和问题。主要是：经济结构性矛盾仍然存在，规模优势不明显，大项目、好项目偏少，有的经济指标增幅在全省位次靠后，市、县属工业经济效益有待提高；交通瓶颈制约依然突出，农村基础设施建设存在不少薄弱环节，抵御自然灾害能力较弱；部分县、乡财政困难状况没有得到根本转变，农民增收难度加大，就业和再就业形势严峻，部分群众的生活仍然比较困难；社会事业发展与人民群众的需求还不适应，农村公共卫生体系比较薄弱，社会治安、安全生产形势不容乐观，城市管理、环境保护和生态建设需要进一步加强；政府职能转变相对滞后，机关工作效率和服务质量有待提高，消极腐败和不正之风不同程度存在，经济发展环境不够宽松，等等。对这些问题，我们将高度重视，采取切实有效的措施努力加以解决。

　　二、立足开拓创新，扎实做好2004年工作

　　今年政府工作的总体要求是：以邓小平理论和“三个代表”重要思想为指导，深入贯彻党的十六大、十六届三中全会和中央经济工作会议精神，落实省委七届六次全会、市委六届八次全会和省、市经济工作会议部署，坚持以人为本，树立全面、协调、可持续的发展观，保护好、引导好、发挥好各方面的积极性。认真实施工业立市、生态兴市、科教强市战略，突出发展县域经济、做大做强做优中心城市和振兴老工业基地，强化项目带动，加快产业发展，着力改革创新，扩大内外开放，改善人民生活，统筹协调推进，努力实现国民经济持续快速协调健康发展和社会全面进步。

　　2004年全市经济和社会发展的预期目标是：全市国内生产总值增长9.5%；全社会固定资产投资增长13%；社会消费品零售总额增长9.5%；外贸出口增长5%；实际利用外商直接投资增长7%；地方级财政一般预算收入增长10%；城镇居民人均可支配收入增长5.5%，农民人均纯收入增长4.5%；城镇登记失业率控制在6%以内；人口自然增长率控制在7‰以内。

　　为实现上述目标，必须扎实做好以下九个方面工作：

　　（一）着力抓好特色产业，切实增加农民收入

　　培育做大特色农业。保护和提高粮食生产能力，建立粮食种子分级储备制度，重点发展优质稻和专用粮，增加粮食储备，完善粮食安全预警应急机制，保障粮食安全。围绕构筑“两带一圈”的县域经济发展格局，以培育特色产业为重点，放手发展各具特色的生态效益型农业，壮大果茶、畜禽、食用菌、苗木花卉等产业。坚持市、县联手，培育肉牛、肉羊、特色水产规模养殖和烟草、笋竹、人工培育红豆杉、建莲深度加工等有发展潜力的七个特色产业，努力扩大规模、提升档次，尽快形成特色、打响品牌。

　　积极推进农业产业化。把培育龙头企业作为农业产业化、工业化的突破口，依托资源上项目，立足原料办工业，培育、壮大一批产值上亿元的龙头企业。以龙头企业为载体，建设区域化、规模化的特色产业基地，推进农产品深度加工和系列开发，延伸农业产业链。依托龙头企业，发挥流通企业、购销大户和农民经纪人的作用，建立农产品营销网络，搞活农产品流通。引入各类主体，参与省、市级现代化农业示范园区建设，完善基础设施，创新经营机制，提高科技含量，拓展农业产业化发展空间。

　　提高服务“三农”水平。推进农科教结合，健全良种引进、示范、繁育、推广体系，总结、推广和深化“农业155”、“村会协作”等经验和做法，扶持发展农村合作经济组织，推进农业科技信息服务网络和服务体系建设。建立健全农业标准化体系和农产品检验检测体系，重视禽流感等重大疫情的预防，做好动植物防疫检疫工作，发展无公害食品、绿色食品和有机食品。增加各级支农资金和农业基本建设投资，探索对种粮农民实行直接补贴方式。开展水利建设年活动，加快病险水库除险加固和水毁工程修复，推进渠系配套和节水灌溉，开展乡村供水工程、山地水利设施和堤防建设。加大农村公路建设力度，计划建设农村公路800公里。完善农村供排水、供电等设施，抓好沼气工程建设，改善农业生态环境。

　　拓宽渠道增加农民收入。贯彻中共中央、国务院《关于促进农民增加收入若干政策的意见》，把增加农民收入作为农业、农村工作的中心任务和基本目标，发挥政策对农民增收的导向作用，逐步建立促进农民收入持续增长的长效机制。挖掘农业和农村内部增收潜力，推进农业结构调整，发展效益农业、创汇农业，扩大就业容量，实现农业增效、农民增收。引导农民从事农村二、三产业，发展乡镇企业和个私经济，参与基础设施、重点工程建设，增加非农收入。拓展外部增收空间，加强对农民工的职业技能培训，开展山海劳务合作，有序组织劳务输出。切实减轻农民负担，防止涉农负担案（事）件的发生。

　　（二）着力抓好产业集群，调整优化工业结构

　　积极培育产业集群。把培育产业集群作为实施工业立市的重要内容，作为发展县域经济和振兴老工业基地的切入点，作为深化项目带动的发展方向。提升传统优势产业发展产业集群，采用高新技术、先进适用技术改造传统产业和老工业基地，支持中央、省属企业新上项目、开发后续产品，发展配套产业，不断提高冶金、化工、纺织、林产加工、建材等产品的技术含量和附加值。紧跟全省产业分工发展产业集群，重点参与全省机械装备制造业基地建设和汽车产业发展，以厦工集团三重公司、永安轴承有限公司等企业为重点，延伸路面机械、环保机械、工程机械等产业链，培育橡塑机械、数控机床等设备制造业，积极发展汽车零部件产业，逐步形成核心产业体系。围绕生态工业发展产业集群，做大生物制药、绿色食品等生态效益型工业，发展光机电仪一体化、新型材料、环保等高新技术产业，培育工业经济新增长点。

　　增强技术创新能力。抓好70个投资千万元以上技改项目和20项重点技术创新项目的实施，争取一批项目列入国家“双高一优”和省技改重点项目盘子。以30家制造业信息化试点示范企业为重点，通过设计、过程、管理、商务数字化，提升产业技术水平。深化产学研合作，促进企业与高校、科研单位对接，加速科技成果转化。支持“三重”牌压路机等省名牌产品争创国家名牌产品，推动“金三明”烟叶等产品争创省级名牌产品，促进现有35个省名牌产品开展品牌经营，增强创立自主知识产权、国产知名品牌的能力。推进企业管理创新，采取内部挖潜、产品开发、市场开拓等措施，提高工业运行质量和效益。贯彻《中小企业促进法》，加强服务体系建设，放开搞活中小企业。

　　（三）着力抓好项目带动，不断增强发展后劲

　　发挥重点建设对投资的支撑作用。今年安排市级重点项目60项，其中在建重点25项、预备重点20项、重点前期15项，总投资397亿元，年度计划投资46亿元。加快高速公路、普通公路、农村公路路网建设，京福高速公路三明段一期工程确保国庆节前通车，二期工程路基基本完成，泉三高速公路三明段控制性工程动工建设，继续推进三明至宁化、永安至武平高速公路项目前期工作，改造、新建国道和省道100公里以上。抓紧街面水电站等一批大中型水电项目的施工，推进火电项目建设，优化水火电比例，加快区域电网建设与改造，做好与省网、华东网连接线扩容改造的规划和实施，推进电力体制改革，促进电力工业发展。

　　强化项目策划与推介。围绕国家、省里产业规划，重点开发关联度大的项目尤其是产业集群项目，并对现有储备项目进行前期深化、滚动开发。加大项目推介力度，依托省“6.18”项目成果交易会等平台，推动项目对接；举办京福高速公路三明段一期工程通车庆典暨投资经贸洽谈系列活动，确保高速公路通车之际，一批项目竣工投产、一批项目开工建设、一批项目对外推介。做好项目申报工作，争取国家和省里支持，管好用好国债资金。拓展项目建设空间，按产业布局调整、整合现有工业园区，重点抓好金沙园、尼葛园开发建设，创新体制机制，完善配套设施，加大招商引资力度，促进项目集聚和建设。

　　创新项目工作载体与机制。深化“抓项目、增后劲、促发展”竞赛活动，完善指标体系和考核办法，进一步调动各级各部门的主动性和积极性。改进项目工作方式和方法，重点创新五项机制，即建立公开征集、充分论证的项目筛选储备机制，健全以项目衍生项目、以客商引进客商的项目生成机制，完善体现合力的项目协调促进机制，强化以质量、安全、进度、资金为保障的项目管理机制，形成主动参与、推动项目的激励机制，使项目建设保持持久的动力和活力。

　　（四）着力抓好各项改革，为加快发展提供动力

　　加快国有经济从一般竞争性领域退出。以产权制度改革为突破口，引进有实力的企业和投资者，加快国有企业改组改制，促进混合所有制经济发展。重点对56家市属国有及国有控股工贸企业实行股份制改造、国有股减持或退出、破产重组等改革，用两年左右时间基本完成国有资本从一般竞争性领域退出。一企一策推进建筑业企业改制，提高整体素质和竞争力。规范国有企业改制，促进国有产权有序流转。健全国有资产管理体制，探索国有资产监管和经营的有效形式，确保国有资产保值增值。

　　大力发展非公有制经济。把非公有制经济作为发展县域经济和振兴老工业基地的生力军，作为推进经济体制完善、运行机制创新和政府职能转变的有效途径。落实加快非公有制经济发展的政策措施，鼓励非公有制经济进入法律法规未禁入的行业和领域，并在投融资、财税、土地使用、劳动保障等方面享有平等待遇。采取合作、联营、入股等方式，促进民间投资增长。支持成立商会、行业协会，开展商会招商、商会融资，发展商会经济。推动“四个一批”私营企业和“小巨人”企业体制创新、联合重组，实现规模和集约经营。建立教育培训、政策咨询等服务平台，加大对融资担保的扶持力度，为非公有制经济发展提供有效服务。

　　完善农村各项改革。推进农村税费改革，及时研究新情况、解决新问题，搞好配套改革，确保农民负担减轻后不反弹，确保乡镇机构和村级组织正常运转，确保农村义务教育经费正常需要。改革和完善征地制度，依法及时给予农民足额补偿。在保持土地承包关系长期稳定的基础上，按照“依法、自愿、有偿”的原则，引导土地承包经营权流转。进一步深化集体林权制度改革，力争全市完成改革任务90%。深化粮食流通体制改革、水利工程管理体制改革，推进供销社所属企业整合、重组。实施《福建省乡镇工作纲要（试行）》，创新乡镇管理体制，促进乡镇经济社会发展。

　　推进财政管理体制改革。创新财政机制，培植财源，实现财政收入持续稳定增长。坚持依法征税，建立健全科学、高效的征管机制，确保各类税款及时入库。认真实施新的市、区财政体制，调整完善县（市）对乡镇的财政体制，壮大村集体经济实力。加大支出结构调整力度，保稳定、保运转、保安全、保改革、保重点，增加财政对社会公共事业的投入。完善部门预算编制、国库管理制度、政府采购制度改革，强化“收支两条线”管理，严肃财经纪律，加强财政、审计监督。积极稳妥办好、办实各项事业，严格举债审批，严禁超财力办事，确保收支平衡。

　　深化行政管理体制改革。改善政府经济调节和市场监管职能，强化社会管理和公共服务职能，增强提供公共服务的能力。贯彻实施《行政许可法》，继续清理市级行政审批事项和相关的规范性文件，依法清理行政许可实施机关。深化行政审批制度改革，创新行政管理方式，提高审批效率。积极稳妥推进事业单位改革，逐步建立政事职责分开、单位自主用人、人员自主择业、政府依法管理、配套措施完善的事业单位分类管理体制。

　　（五）着力抓好招商引资，拓展发展空间

　　有效开展引资工作。围绕振兴老工业基地、延伸产业链、发展产业集群，利用“9.8”投洽会等开展引资工作，继续举办工业、农副产品加工业等行业招商活动，探索中介招商、网上招商等形式，引导客商投资特色农业、重点产业、服务业和开发区，推动现有企业增资扩产，实现二次招商。加大签约项目的跟进落实力度，提高项目的履约率、到资率。发挥外事、侨、台和驻外机构的作用，共同做好联内引外工作。

　　继续扩大外贸出口。调整出口商品结构，鼓励企业出口农产品、机电产品等退税率不变或下调幅度小的产品，指导造纸、矿产品加工等退税率下调幅度大的企业发展加工贸易，促进出口稳步增长。完成6家市属并启动县属国有外贸企业改组改制，鼓励国有、私营、外资企业扩大出口，促进出口主体多元化。推进“大通关”建设，提高通关速度和效率。发展对外劳务合作，引导有条件的企业特别是私营企业，拓展境外生产和营销。

　　深化山海协作和区域合作。主动融入海峡西岸经济区建设，积极承接沿海地区产业和企业转移，推动34家省级山海协作示范企业和骨干企业深化合作，加快建设连接山区和沿海发达地区的经济产业带。主动接受对口帮扶，促进优势互补和产业协作，引导机械、农产品加工等企业在产业对接中加快发展。加强区域协作，逐步推进与长江三角洲、珠江三角洲的产业、市场、技术对接，增强区域经济实力。

　　（六）着力抓好商贸旅游，提高服务业发展水平

　　坚持搞活流通与拓展消费并举。推进市场体系建设，加快建设汽车城等专业特色市场，引进国内外知名流通企业改造传统专业市场，抓好连锁经营、物流配送、电子商务等现代流通业发展，建立与地产品主销区、大型超市、物流配送中心连接的信息网络平台。发展会展经济，积极培育餐饮等消费热点，拓展居民住房、汽车等大宗商品的消费。鼓励和推广信用消费，促进个人消费贷款，拓宽市场消费空间。培育和保护农村购买力，鼓励大中型商贸企业发展农村营销网点，进一步扩大农村消费。

　　培育壮大旅游产业。构筑生态旅游胜地，整合优化旅游资源和旅游产品结构，加快建设国家级、省级重点景区基础设施，打造生态旅游、都市休闲旅游、寻根谒祖等旅游精品，支持大金湖申报世界地质公园。办好“中国（三明）百姓生活游”主题活动，打响市区周边旅游圈、大金湖、客家祖地三大品牌，联合区内外大旅行社、大景区，拓展省内外重点客源市场。改善旅游交通条件，完善旅游接待设施。加强旅游行业管理，加快培养高素质的旅游经营管理与服务人才，提高企业经营管理水平。

　　大力发展新兴服务业。培育文化娱乐、教育培训、体育健身等产业和物业管理、家政服务等社区服务业，规范发展会计咨询、资产评估、法律咨询等中介服务。加大金融对地方经济发展的支持力度，改进和拓展金融服务，采取有效办法处置不良资产。推进农村信用社改革与发展，推广农户小额信用贷款和农户联保贷款。支持金融改革与发展，加强金融监管，维护金融债权，化解金融风险。改革投融资体制，扩大直接融资。增强全社会的信用意识，提高企业信用水平，建立健全信用监管和失信惩戒机制。

　　（七）着力抓好城乡建设，积极推进城镇化进程

　　拓展城市发展空间。根据“南拓北扩西进”的思路，拉开市区发展构架，南面抓好台江、富兴堡两个片区的规划、开发，北面加快贵溪洋片区和瑞云新区的规划、建设，西面以入园道路建设为突破口，抓紧小蕉工业园区规划、开发，逐步扩大市区建成区面积。按照“控制中心、拓展两翼、开发西江滨路沿线”的要求，推进房地产业发展，严格控制三明市区中心区的新房地产开发项目，重点开发京福、泉三高速公路三明连接线入口处周边的贵溪洋、台江等片区，并围绕西江滨路沿线的开发，加快龙岗等片区的建设，有计划地实施经济适用住房建设，促进市区房地产业健康、有序发展。抓好城市道路建设与改造，加快重要节点的设计和建设，继续实施市区园林绿化、沿河景观与夜景工程建设，挖掘城市文化内涵，提高城市品位。探索城市联盟的发展路子，打破行政区划界限，以京福高速公路三明连接线和沙溪为主轴，以金沙园的开发建设为突破口，促进城市向外拓展，稳步推进城市一体化进程。

　　推进以县城为重点的城镇建设。编制和完善县域城镇体系规划，分步实施老城区改造和新区开发，加快省、市级住宅试点小区建设，因县制宜挖掘生态、文化等特色，提升县城档次。稳步推进小城镇建设，突出抓好14个省定重点镇和京福高速公路互通口所在镇规划建设，以及20个省级村镇住宅试点小区工作，充分发挥示范带动效应。贯彻落实有关户籍改革的政策措施，通过产业发展、项目建设和园区开发集聚城镇人口。

　　加强城市经营和管理。健全完善土地收购储备运行体制和机制，加强政府对土地一级市场的垄断，推行土地招标拍卖、挂牌竞价，增加土地级差收益。积极探索新的融资运作方式，吸引各类主体参与城市基础设施建设、经营，对道路及其两侧土地、房地产进行捆绑开发建设，租赁、拍卖供水、污水处理和城市公交线路等特许经营权，以及道路设施广告权和街道、公园等冠名权，以灵活的体制、机制和政策集聚资金。建立健全城市管理工作机制，依托城市管理联动中心和城管队伍，突出长效管理和社区管理，开展脏、乱、差等专项整治和城市环境综合治理，提高城市管理水平。

　　（八）着力抓好科技教育，促进社会事业协调发展

　　做好科技、人才和教育工作。实施科教强市，加快国家技术创新工程区域试点城市、沙溪沿岸星火技术产业带建设，构筑生产力促进中心、高新技术创业服务中心、金沙园中试基地、星火信息网四个平台，加强产业科技工作，促进技术创新示范（试点）企业、民营科技企业发展。创建科普教育示范基地，做好科学普及工作。贯彻党中央、国务院《关于进一步加强人才工作的决定》，建立健全人才评价和使用机制，多形式、多渠道培养和引进人才。加大引智工作力度，建立人才柔性流动机制。调整学校布局结构，抓好“两基”巩固提高，加强对农村义务教育的管理，全面推进“双高普九”，办好学前教育，加快发展普通高中教育，培育和扩大优质高中。继续深化基础教育课程改革，提高教学质量和办学水平。计划投资9800万元，分3年完成19.72万平方米农村中小学D级危房改造任务。加快发展高等教育，推进三明学院建设。巩固发展职业与成人教育，办好国家、省级重点中等职业学校，筹建职业技术学院，实施高中级技工培训工程。创新办学体制，鼓励、支持社会力量办学。

　　加快卫生、文化等社会事业发展。毫不松懈抓好非典预防工作，加快构筑公共卫生安全防护网，今明两年投资1000万元，推进疫情信息网络体系、疾病预防控制体系、突发公共卫生事件医疗救治体系、卫生执法监督体系建设。深化城镇医疗卫生体制改革，改善农村医疗卫生条件，探索建立农村大病统筹制度，对贫困农民实行医疗救助。广泛开展爱国卫生运动，巩固提高国家卫生城的成果。大力推进文化精品创作，丰富群众文化生活，抓好文物保护与利用，繁荣和净化文化市场。广泛开展全民健身活动，努力提高竞技体育水平。把握正确的舆论导向，提高广播电视节目制作质量，加快广播电视数字化建设步伐。

　　促进人与自然和谐发展。扎实开展创建计生优质服务先进县（市、区）活动，加强基层计生协会“双创”、“两为”工作，集中力量解决出生人口性别比偏高、社会抚养费征收难、流动人口漏管等突出问题。严格保护耕地，保持耕地占补平衡。加强矿业权市场管理和建设，培育和规范探矿权、采矿权市场，做好地质灾害防治工作。实施生态兴市，落实市长环保目标责任制，抓好大气污染治理、水环境整治和固体废弃物处理，严格控制主要污染物排放总量，完善环境监测监控体系，加强国家级、省级自然保护区和生态示范区建设，改善城乡环境综合质量。

　　加强精神文明和民主法制建设。大力弘扬和培育民族精神，加强思想道德建设，开展群众性精神文明创建活动，巩固创“五城”成果，努力争创全国文明城市，进一步提高城乡文明程度。深入实施《福建省城市社区建设纲要（试行）》，改善基础设施条件，健全服务保障体系。贯彻落实《福建省社会治安综合治理纲要（试行）》，围绕创建“平安三明”，加强社会治安防控体系建设，建立健全经常性的严打工作机制，依法打击“六合彩”等赌博活动。加强国家安全工作，防范和处理“法轮功”等邪教组织的非法活动。高度重视群众来信来访，完善矛盾纠纷排查组织网络和调处工作机制，积极预防和妥善处置突发群体性事件。整顿和规范市场经济秩序，治理“餐桌污染”，建设“食品放心工程”，健全市场监管体系。进一步落实安全生产责任制，继续开展“查隐患、抓整改、保安全”的专项整治，防范重大安全事故的发生。建立应急反应机制，完善和落实各级经济社会紧急情况应急处置工作预案。深入推进“四五”普法，提高公务人员的法律素质和依法办事水平，增强全民法律意识和法制观念，推进依法治市进程。认真实施国务院《法律援助条例》，拓展和规范法律服务。不断完善民主决策程序，认真听取民主党派、工商联、无党派人士和人民团体的意见，建立和完善专家咨询制度、社会公示和社会听证以及决策责任制度。促进基层民主建设，健全村（居）民自治制度和企事业民主管理制度。加强国防教育，深入开展“双拥”活动，积极支持驻明部队和武警部队建设，推动国防动员、人民防空和民兵预备役建设。抓好民族、宗教、档案、地方志、气象、水文、防震减灾等工作，加快妇女、儿童、老龄和残疾人事业的发展，做好关心下一代工作。

　　（九）着力抓好社会保障，改善城乡居民生活

　　继续做好就业和再就业工作。健全各级政府促进就业目标责任制，实行积极的就业政策，大力发展劳动密集型产业和非公有制经济，争取新增城镇就业27000人，净增4000人。全面落实再就业扶持政策，实施再就业援助，促进“4050”群体再就业。完善和推广就业、培训、保障、维权相结合的劳务派遣组织，大力推进多种形式就业。逐步建立统一、规范的城乡劳动力市场，完善街道（乡镇）、社区就业服务平台，加强职业技能培训，培育市场导向的就业机制。

　　进一步完善社会保障体系。扩大养老、失业保险覆盖面，确保离退休人员基本养老金和失业人员失业金按时足额发放。探索机关事业单位人员养老保险制度的改革办法，加快推进企业退休人员社会化管理。落实党和国家关于老干部的各项政策，保证他们安度晚年。贯彻落实国务院《工伤保险条例》，深化医疗保险制度改革，继续扩大医疗保险覆盖面，完善多层次的医疗保险体系。

　　关心困难群众生活。大力支持革命老区、贫困山区和少数民族地区的基础设施建设，加大扶贫开发力度，重点抓好32个省级扶贫重点村建设，改善群众的生产生活条件。继续实施“造福工程”，加快农村旧村改造、新村建设及城镇危旧房改造。增加城乡居民收入，防止收入差距扩大。做好社会福利、社会救济、优抚安置和社会互助等工作，妥善安排困难群体的生活。做好城市居民最低生活保障工作，推进农村居民最低生活保障制度的实施，将农户年人均收入低于1000元的8.08万农村贫困人口全部纳入低保范围，争取实现应保尽保。按照群众受益、量力而行的原则，继续实施一批为民办实事项目。

　　三、着眼营造发展环境，进一步加强政府自身建设

　　树立充满生机和活力的政府形象，不断提高行政水平和服务质量，是营造良好发展环境、加快三明经济社会发展的关键所在。我们必须坚持在市委的领导下开展工作，贯彻落实市委的各项部署和决定；依法接受人大及其常委会的监督，主动报告工作；积极支持人民政协履行政治协商、民主监督、参政议政的职能，主动通报情况；重视人大代表建议、意见和政协提案办理工作，主动争取各方面支持，不断提高管理社会、促进发展、服务人民的能力。

　　（一）建设以人为本、为民谋利的政府。强化“民本”意识、“公仆”观念，在政府工作中更多体现人文关怀，更多关注全体社会成员的公共利益，搞好公共管理和公共服务，让群众更直接地享受发展成果，得到更多实惠。了解群众呼声，关心群众冷暖，真正弄清群众在想什么、干什么、盼什么、怨什么，忧民所忧，急民所急，民有所呼、我有所为，把群众关心的小事当作大事办，把群众关注的热点作为重点抓。统筹兼顾各方面群众的利益，做大多数群众赞成的事，办大多数群众受益的事，任何时候都不能与民争利。把改革的力度、发展的速度和社会可承受的程度统一起来，坚持按政策办事、依法办事，努力减少矛盾、化解矛盾。

　　（二）建设奋发有为、敢于负责的政府。坚持解放思想、实事求是、与时俱进，冲破影响发展的思想观念、束缚发展的陈规陋习、制约发展的体制弊端，在加快发展中解放思想，在解放思想中统一思想。坚持以发展为己任，敢闯敢试、敢抓敢管、敢作敢为，看准的事盯紧抓实，探索中的事勇于突破，有争议的事大胆尝试，以发展树形象。大力营造创新氛围，支持创造、支持创业，让想干事的有机会，能干事的有舞台，干成事的有地位。树立正确的政绩观，坚持对党和人民负责，对历史和现实负责，作决策要科学，定一条是一条，条条算数；做事要到位，做一件成一件，件件落实；承诺要有度，说一项是一项，项项兑现。

　　（三）建设高效运作、求真务实的政府。高度重视公务员的思想、政治、作风、业务建设，加强理论武装，加快知识更新，不断提高队伍的综合素质。政府工作千头万绪，关键在落实，核心是效率，要落实效能告诫、绩效考评等制度，深化机关效能建设，多一点实干举措，多一点有效服务，少一点形式主义，少一点繁文缛节。大力弘扬求真务实精神，大兴求真务实之风，进一步贴近基层，到一线了解情况，到一线建立互信，到一线推动工作，以实际行动赢得群众的理解和支持。

　　（四）建设依法行政、清正廉洁的政府。依法规范政府行为，严格行政执法责任制和执法过错追究制，加强行政复议工作，做到严格执法、公正执法、文明执法，防止和克服行政“不作为”和行政“乱作为”。坚持标本兼治、综合治理，加强政风建设，纠正部门和行业不正之风，落实反腐倡廉“六个机制”，推行政务公开，从源头上防治腐败。继续严肃查处违纪违法案件，严厉打击和清除腐败分子。全体公务人员特别是领导干部要遵守政治纪律、组织纪律、经济工作纪律和群众工作纪律，时刻牢记“两个务必”，做公道正派之人、诚实厚道之人、谦虚谨慎之人、清正廉洁之人，切实履行好党和人民赋予的神圣职责。

　　各位代表，新的一年政府工作任务艰巨、责任重大。让我们紧密团结在以胡锦涛同志为总书记的党中央周围，高举邓小平理论伟大旗帜，全面贯彻“三个代表”重要思想和党的十六大精神，在省委、省政府和市委的领导下，紧紧依靠全市人民，万众一心，攻坚克难，开拓前进，为再创三明改革开放和现代化建设新业绩而努力奋斗。
